# Molecular Research on Alzheimer’s Disease

**DOI:** 10.3390/biomedicines11071883

**Published:** 2023-07-03

**Authors:** Lorenzo Falsetti

**Affiliations:** Internal and Subintensive Medicine Department, Azienda Ospedaliero-Universitaria delle Marche, 60131 Ancona, Italy; lorenzo.falsetti@ospedaliriuniti.marche.it

Alzheimer’s disease (AD) is the most common form of dementia worldwide. Despite its prevalence and incidence, there are few and limited specific treatments for this disabling and progressive disorder. Anti-amyloid therapy effectiveness is still controversial, but it seems to improve quality of life and reduce AD progression in mild or moderate forms, at the cost of potentially serious adverse effects, especially urinary tract infection, nervous system disorders, intracranial hemorrhage, and amyloid-related imaging abnormalities. However, this treatment seems able to reduce the burden of brain amyloid, which represents the final waste product of complex molecular pathways, leading to AD neurodegeneration. The first topic discussed in this Special Issue is related to current and future molecular methods suggested to improve AD diagnosis. Often, patients cannot be selected for specific treatments, studies, or enrolled in clinical trials due to mixed or atypical presentations: in these settings, AD diagnosis should be enriched with cerebrospinal fluid (CSF) biomarker interpretation, which still represent the cornerstone for differential diagnosis in the setting of neurodegenerative diseases, allowing one to differentiate AD from other forms of dementia, such as vascular forms [1]. Moreover, integrating clinical, neuropsychological, and radiological data with the AT(N) biochemical profiling system (amyloid, tau pathology, and neural loss) allows the researcher and the physician to refine AD diagnosis for both research and clinical purposes, allowing one correctly frame the patient, even in atypical clinical presentations. Current molecular research is also proposing novel serum plasma markers, such as plasma phospho-tau-181, that, in the near future, will be adopted to refine AD diagnosis and to predict its progression, without the need for an invasive lumbar puncture for CSF biomarker determination [2]. Still, this novel marker of disease deserves extensive validation: especially, the optimal method of determination should be standardized to determine its exact sensitivity or specificity values. The second topic covered in this issue of Biomedicines, “Molecular Research of Alzheimer’s Disease”, deals with the description of the complex molecular pathways associated with AD pathophysiology. Identifying innovative molecular targets could lead to more effective treatments to reduce both the incidence and the progression of this neurodegenerative disease. Most AD cases are not inherited and become clinically evident in elderly, multicomorbid subjects. In this setting, the pathophysiology of AD neurodegeneration seems complex and seems to be associated with the disruption of several molecular pathways, compromising the function of neuronal, glial, and neurovascular units. An increased deterioration of cognitive function has been observed in patients showing a status of comorbidity, considering—among a patient’s associated disorders—vascular risk factors, such as diabetes, hypertension, dyslipidemia, and cigarette smoking, which are associated with neuroinflammation, neurovascular unit dysfunction, and blood–brain barrier breakdown [3]. Evidence suggests that AD and its associated comorbidities share molecular pathways, leading to a faster cognitive decline: one of the most intriguing molecular overlaps between neurodegenerative and systemic diseases is symbolized by diabetes mellitus. Alterations in the insulin signaling pathway and glucose resistance in AD subjects’ brains are common and typical, and AD is commonly referred as to type 3 diabetes mellitus. Insulin resistance translates into a chronic signaling activation of the mechanistic target of rapamycin (mTOR), leading to blood–brain barrier dysfunction, tau hyperphosphorylation, and amyloid plaque formation. Thus, the insulin receptor could represent a potential target to improve neurogenesis. Some molecules, such as amarogentin, seem able to interact with this receptor, representing potential candidates for future clinical studies [4]. Other classes of newer drugs, such as SGLT-2 inhibitors [5] and sestrins [6], could be considered to reduce mTOR activity, acting after the insulin receptor cascade and slowing neurodegeneration. Sestrins seem able to also act in other commonly disrupted pathways in AD, such as, for example, by improving antioxidation and adjusting autophagy. Several other genes involved in oxidative stress and mitochondrial dysfunction have been associated with mild cognitive impairment and AD [7], representing targets of interest for future investigations. Another typical aspect of AD is characterized by neuroinflammation, especially in AD’s later stages [8]. As in most tau-dependent neurodegenerative diseases, the interplay between astrocytes, microglia, and neurons often shift from an early, neuroprotective, tau-clearing phenotype with an exacerbated autophagy-lysosomal pathway to a “loss of function” phenotype, leading to neuronal excitotoxicity, often associated with a neuroinflammatory phenotype, which is related to increased tau pathology, oxidative stress, and increased amyloid deposition [9]. Microglia seem to play a pivotal role in the neuroinflammatory component observed in AD neuropathology. Of note, melatonin and other similar molecules act on neuroinflammatory pathways and seem able to upregulate SIRT1-mediated brain-derived neurotrophic factor with regards to prolonged microglial exposure to Aβ42. This could translate into a reduced expression of inflammatory markers, such as IL-1β and (TNF)-α, with a subsequent downregulation of the proinflammatory pathway, which is mediated by NF-κB [10]. Other molecules, such as curcumin, have been shown to be effective in reducing inflammation, oxidative stress, and the aggregation of amyloidogenic proteins [11]. Albeit interesting, most of the published papers in this issue show evidence at a preclinical stage, and further clinical studies are required to validate and to extend the interesting results collected in this issue of Biomedicines. Despite actual knowledge, more insights into the molecular mechanisms, leading to the amyloid cascade, are still needed to improve diagnostic methods and to to explore novel therapeutic agents acting on different molecular targets of the neurodegenerative cascade of Alzheimer’s disease.
